# Author Correction: Optical and structural properties of Sn and Ag-doped PbS/PVA nanocomposites synthesized by chemical bath deposition

**DOI:** 10.1038/s41598-023-36765-2

**Published:** 2023-06-19

**Authors:** Ali Fatemi, Tavakkol Tohidi, Kazem Jamshidi‑Galeh, Milad Rasouli, Kostya Ostrikov

**Affiliations:** 1 Department of Physics, Azarbaijan Shahid Madani University, Tabriz, Iran; 2 Northwest Research Complex (Bonab), Radiation Applications Research School, Nuclear Science and Technology Research Institute (NSTRI), Tehran, Iran; 3 Department of Physics, Kharazmi University, Tehran, Iran; 4 Department of Physics, Science and Research Branch, Islamic Azad University, Tehran, Iran; 5 School of Chemistry and Physics and QUT Centre for Materials Science, Queensland University of Technology (QUT), Brisbane, Australia

Correction to: *Scientific Reports* 10.1038/s41598-022-16666-6, published online 28 July 2022

The original version of this Article contained errors in Figure 1. During preparation of Figure 1 some of the data has been inappropriately superimposed. As a result, in Figure 1A a section of the spectrum for the “0.6 μM Sn” condition overlapped with a corresponding section in the spectrum of the “0.3 μM Sn” condition. A section of the spectrum for the “0.8 μM Ag” condition overlapped with corresponding sections in the spectra for “0.4 μM Ag” in Figure 1B, and “0.6 μM Sn” and “0.3 μM Sn” conditions in Figure 1A. The original incorrect Figure 1 and accompanying legend appear below.

**Figure 1:**
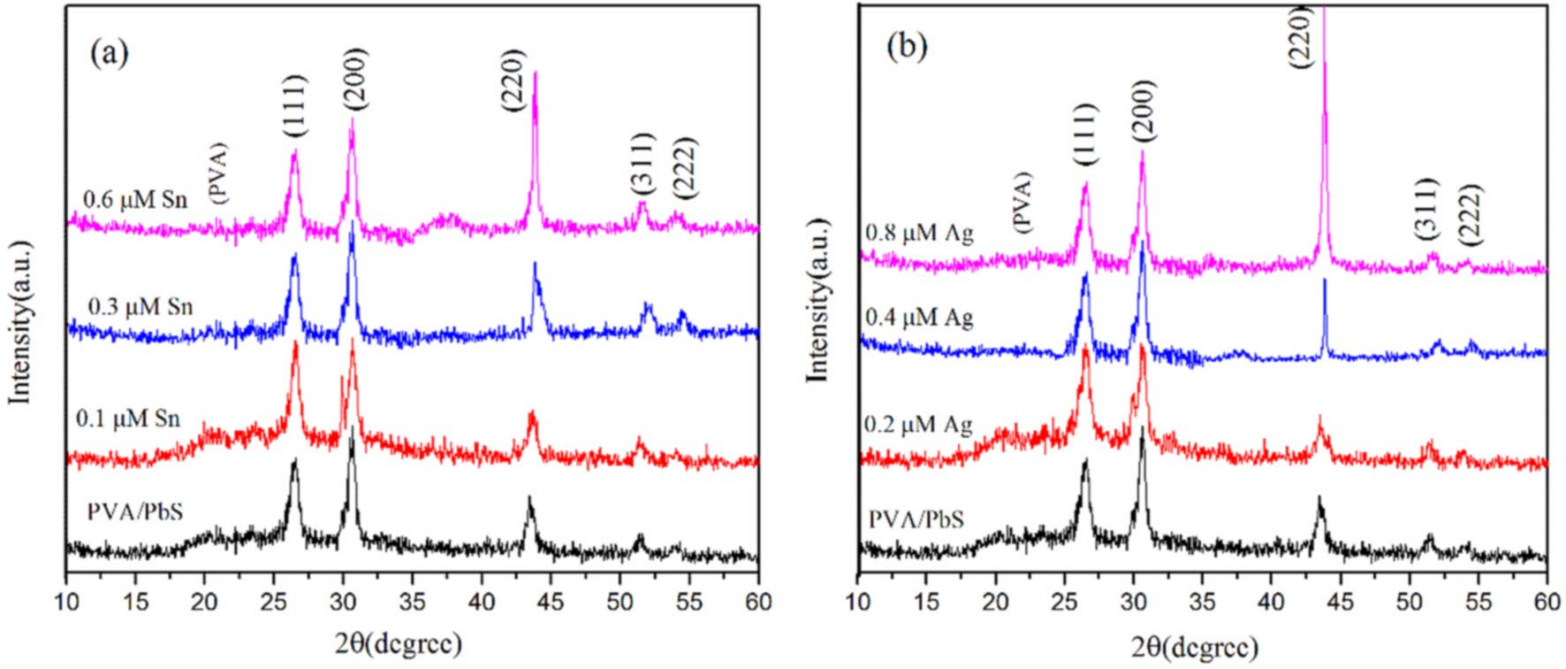
(**a**) XRD spectrum of undoped and Sn doped and (**b**) XRD spectrum of undoped and Ag-doped PbS/PVA nanocomposites.

The original Article has been corrected.

